# Small LLMs can be good coldstart recommenders

**DOI:** 10.3389/frai.2026.1705245

**Published:** 2026-03-20

**Authors:** Joseph Noel, Christopher Monterola, Daniel Stanley Tan

**Affiliations:** 1 Aboitiz School of Innovation, Technology and Entrepreneurship, Asian Institute of Management, Makati, Philippines; 2 Faculty of Science, Open Universiteit Nederland, Heerlen, Limburg, Netherlands

**Keywords:** coldstart recommendations, large language models, machine learning, PEFT, recommendation systems

## Abstract

Large Language Models (LLMs) have revolutionized the Artificial Intelligence (AI) field since the launch of ChatGPT in 2022. Since then, increasingly larger models have been released such as ChatGPT-4o having over 175 billion parameters, Llama 3.1 with 405 billion parameters, and PaLM with 560 billion parameters. However, LLMs of these sizes are no longer feasible to run easily outside of the largest research labs and organizations due to the extremely large amount of GPU compute required for both training and inference. More recently, research effort has been done to create smaller LLMs which can still perform relatively well compared to much larger models. Research has also been done to apply LLMs for domain-specific use cases such as recommendation systems via prompt engineering and fine-tuning. In this paper we combine the two research fields and fine-tune two small LLMs (2 billion parameters or less) for the sequential recommendation task. We find that fine-tuned small LLMs still perform as well and can even be better than standard sequential recommendation baseline models such as GRU4Rec and SASRec, especially in the coldstart setting.

## 1 Introduction

Pre-trained Large Language Models have been useful as foundation models which can then be customized for downstream tasks either via prompt engineering or via fine-tuning. They have been customized to domains such as music (Agostinelli et al., 2023), healthcare (Meng et al., 2024), education (Ma et al., 2023; Khalid et al., 2021; Haruna et al., 2017), and forecasting (Jin et al., 2024). LLMs have also been applied to the recommendation domain in numerous works (Sanner et al., 2023; Bao et al., 2023; Harte et al., 2023; Wei et al., 2024), taking advantage of their pre-trained learned representations and the expressivity of natural language. However most research of LLMs for recommendation systems make use of state-of-the-art LLMs with over 100 billion parameters. Training and running LLMs of these sizes are intractable for all but the largest and best funded organizations due to the extremely large amount of GPU compute required for both training and inference.

Small LLMs are becoming an active field of development (Wan et al., 2024) due to their cheaper compute cost and they have become more capable over time. These models are small enough that they can be fine-tuned and run using off-the-shelf GPUs which are more readily available to everyone.

In this paper we explore fine-tuning small LLMs with 2 billion parameters or less for the recommendation domain. We use Low-Rank Adaptation (LoRA) to fine-tune two small LLMs, Danube-1.8B (Singer et al., 2024) and Gemma-2B (Team et al., 2024), and evaluate them on two standard recommendations datasets, MovieLens10M (Harper and Konstan, 2015) and Yoochoose-clicks (Ben-Shimon et al., 2015). We find that the fine-tuned LLMs are able to adequately learn to do sequential recommendation, and are able to beat the baseline recommendation models in the coldstart setting. To the best of our knowledge this is the first work on fine-tuning small LLMs for the sequential recommendation domain in a coldstart setting.

## 2 Background and related work

### 2.1 Recommendation systems

Let *U* be the set of users and *X* be the set of items. Recommendation systems use machine learning algorithms to predict a user-item rating *R*(*u, x*) for all users *u*∈*U* and all items *x*∈*X*.

Different classes of recommendation system models have been developed over the years, such as Content-based Filtering (Lops et al., 2019), Collaborative Filtering (Zhang et al., 2014; Salakhutdinov and Mnih, 2007; Linden et al., 2003), and Sequential Recommendation (Wu et al., 2019; Li et al., 2017; Liu et al., 2018; Yu et al., 2020; Hidasi and Karatzoglou, 2018; Wang-Cheng Kang, 2018). Availability of past user preference information is a concern as model training is mainly done on previous user-item interactions such as explicit rating scores and implicit activities such as item clicks or views.

#### 2.1.1 Sequential recommendation

A common formulation of the recommendation problem models the data as a sequence of user-item interactions. In sequential recommendation, let *X* be the set of items to be recommended, and *x*_1:*t*_ = *x*_1_, *x*_2_, ..., *x*_*T*_ be the the sequence of past user-item interactions where *x*_*i*_∈*X* is the user-item interaction at timestamp *t*. A sequential recommendation model can be a multi-class classifier where, given the interaction sequence *x*_1:*t*_, the model tries to predict the next item in the sequence *x*_*t*+1_. The model output can be a ranked list of items with classification logits ${\text{y}}_{t+1}=\left[y_{1},y_{2},...,y_{n}\right]\in {\mathbb{R}}^{n}$ where *n* = |*I*| is the number of possible items. The final recommendation list at timestamp *t*+1 are the top-*k* items from **y**_*t*+1_.

#### 2.1.2 Coldstart recommendation

The coldstart problem is one of the fundamental research problem in the field of recommendation systems and numerous research efforts have been dedicated to solving it (Gope and Jain, 2017). Because traditional recommendation models rely on past user-item interactions, new users and items won't have had enough historical information to make accurate recommendation predictions on. New items in particular may be disadvantaged and won't be recommended at all due to popularity bias which can favor older items (Noel et al., 2024; Abdollahpouri et al., 2017).

Popular methods devised for handling the coldstart problem include incorporating user and item attributes in the model training (Gantner et al., 2010; Burke, 2007), hybrid content-based and collaborative filtering algorithms (Schein et al., 2002; Stern et al., 2009), user classification (Lika et al., 2014), cross-domain recommendation (Kang et al., 2019; Man et al., 2017; Omidvar and Tran, 2023), and novel objective functions and regularization (Wei et al., 2021; Abdollahpouri et al., 2017; Kuznetsov and Kordík, 2023).

With the advent of LLMs, new methods for data augmentation (Wei et al., 2024) and initial preference elicitations (Sanner et al., 2023) have also been explored for coldstart recommendations.

### 2.2 Large language model recommendation systems

Numerous works have previously explored the use of language models for recommendation. One such use is as a single unifying model architecture that can handle different recommendation problems such as sequential recommendation, ratings prediction, and review summarization (Harte et al., 2022). Others have used bi-directional encoders pioneered in BERT (Devlin et al., 2019) for recommendation (Zhang et al., 2019; Chen et al., 2019).

The advent of pre-trained Large Language Models has brought about numerous research investigating their applicability for recommendation. Most applications have focused on their usefulness on the coldstart problem. LLMs from OpenAI have been used for data augmentation to handle the common coldstart and data sparsity challenge in recommendation systems (Wei et al., 2024). OpenAI's LLM text embeddings have also been used to initialize BERT4Rec (Zhang et al., 2019) item embeddings and have been found to improve its performance (Harte et al., 2023). Google's PaLM (Chowdhery et al., 2023) was used to investigate solely using prompt-engineering for recommendation and found it was competitive in near cold-start settings (Sanner et al., 2023). Facebook's Llama (Touvron et al., 2023a) was fine-tuned for a binary recommendation problem and performed well in a few-shot setting which is similar to coldstart (Bao et al., 2023).

The above examples all use state-of-the-art and extremely large language models with the order of hundreds of billions of parameters. Running these LLMs locally will require extremely large amounts of compute which may not be feasible for most organizations. We explore two options for scaling down the requirements of using LLMs for recommendation: Smaller large language models and parameter-efficient fine-tuning.

#### 2.2.1 Small large language models

Recently, research has been done on the creation of smaller large language models which can still perform competitively with the much larger state-of-the-art LLMs (Wan et al., 2024; Sheik et al., 2024). Smaller models are easier to fine-tune and will have cheaper inference costs when deployed in real-world production use-cases (Singer et al., 2024; Team et al., 2024; Zhang et al., 2024). We note that the term “small” here is relative and applied specifically to LLMs which have less than 2 billion parameters, which can still be quite large when compared to more traditional machine learning models.

#### 2.2.2 LLM fine-tuning

Fine-tuning is a commonly used technique in deep learning to take advantage of pre-trained models and adapt them for other domains (Jung et al., 2015; Yin et al., 2017; Jaques et al., 2016, 2017; Howard and Ruder, 2018). Due to the extremely large model sizes of LLMs, updating the weights of the entire model is infeasible without having access to a large amount of GPU compute which may be unrealistic for most organizations and researchers. Techniques for parameter-efficient fine-tuning (PEFT) (Ding et al., 2023) needed to be developed.

One of the more popular PEFT methods is Low-Rank Adaptation (LoRA) (Hu et al., 2022). LoRA works by adding a small number of new weight matrices into the model and only these new additions are updated during fine-tuning training. The much smaller number of trainable parameters makes the fine-tuning process faster and more efficient compared to updating all the weights of the entire model. Formally, the learning objective for an LLM can be defined as


$$\begin{array}{l} \underset{\text{Φ}}{\max}\underset{\left(x,y\right)\in Z}{\sum }\overset{\left|y\right|}{\underset{t=1}{\sum }}\log\left(P_{\text{Φ}}\left(y_{t}|x,y_{<t}\right)\right), \end{array}$$

where *x* and *y* are in the input and output tokens of training set *Z*, *y*_*t*_ is the *t*-th token of *y*, *y*_<*t*_ are the tokens before *y*_*t*_, and Φ is the original parameter weights. The number of parameters in Φ will be very large for large language models, therefore LoRA introduces a new set of parameters Θ such that


$$\begin{array}{l} \underset{\text{Θ}}{\max}\underset{\left(x,y\right)\in Z}{\sum }\overset{\left|y\right|}{\underset{t=1}{\sum }}\log\left(P_{\text{Φ}+\text{Θ}}\left(y_{t}|x,y_{<t}\right)\right), \end{array}$$

and where the number of new parameters in Θ will be much smaller than the number of parameters in Φ. Only the parameters of Θ are updated during the LoRA fine-tuning, making this more efficient and less time-consuming than regular fine-tuning.

In the next section we fine-tune small LLMs for the sequential recommendation domain in the coldstart setting using LoRA and show our results.

## 3 Experiments

### 3.1 Dataset

We evaluate our method using two datasets: Yoochoose-clicks (Ben-Shimon et al., 2015), and MovieLens10M (Harper and Konstan, 2015). In MovieLens10M each user-movie rating is considered an interaction, with the actual value of the rating being disregarded. In Yoochoose-clicks we remove duplicate item IDs from the sequences. We also follow a common practice of sampling only a fraction of the data due to its large size (Wu et al., 2019; Li et al., 2017; Liu et al., 2018; Yu et al., 2020).

For both datasets we use the last interaction in the sequence as the prediction target *x*_*t*+1_, and only get the previous 5 interactions as the input sequence. This leaves a limited set of interactions which simulates the coldstart setting, where only a small number of users will have a limited number of interactions with the items. Table 1 shows the number of sequences used for training and testing in our experiments. Only the item IDs are used from the dataset and no other additional user or item features have been utilized.

**Table 1:** Number of sequences used for training and testing.

**Dataset**	**Training sequences**	**Tes Sequencest**
Yoochoose	53,840	13,560
MovieLens	55,902	13,976

#### 3.1.1 LLM fine-tuning dataset

To fine-tune the LLMs we need to convert the datasets into prompts suitable for causal language modeling. Causal language modeling is a text generation workflow which predicts the next token in a sequence of tokens, using only the previous tokens as information. We convert the sequence input and target output into prompts in the following manner shown in Table 2. The final word target output is left out of the test input data and the LLM is prompted for the predicted output.

**Table 2:** LLM fine-tuning dataset converted to prompts.

**Dataset**	**Example**
Training data	*“This user interacted with the following items in the given order: 466, 520, 151, 1408, 1912. The next movie the user will click is 8784”*
Test data	“*This user interacted with the following items in the given order: 832, 1271, 3108, 3252, 224. The next item the user will click is”*
Target output	“*5629”*

### 3.2 Small LLMs

We use two open-source LLM models in our experiments, Danube-1.8B (Singer et al., 2024) and Gemma-2B (Team et al., 2024). Danube-1.8B is a decoder model based on the Llama 2 architecture (Touvron et al., 2023b) with 1.8 billion parameters and trained on 1 trillion tokens. Gemma-2B is decoder model with 2 billion parameters and trained on 3 trillion tokens. A key element in selecting these models is their open-source nature so that their trained model weights are available online^1^^,^^2^ and can be used for further fine-tuning.

We use LoRA (Hu et al., 2022) for efficient fine-tuning of both models for recommendation, using the converted prompts dataset detailed in the previous section. Table 3 shows the number of trainable parameters that were used for fine-tuning and what percentage they were of the original model size. It can be seen that we are updating only a very small fraction (less than 1%) of the number of weights of the original models, which allows us to fine-tune them on relatively dated NVIDIA GeForce GTX 1080 Ti GPUs.

**Table 3:** Number of trainable LoRA parameters and their percentage of the original model size.

**LLM**	**Trainable parameters**
Danube	8,650,752 (0.47%)
Gemma	9,805,824 (0.39%)

#### 3.2.1 Baseline models

We compare against three deep learning sequential recommendation models as baselines, GRU4Rec (Hidasi et al., 2016), SASRec (Wang-Cheng Kang, 2018) and BERT4Rec (Zhang et al., 2019). All models are trained with the Adam (Kingma and Ba, 2015) optimizer using cross-entropy loss. Model hyperparameters were tuned with grid-search and the best results are reported.

The details of their final model architectures are below:

- **GRU4Rec:** 1 embedding layer and 1 GRU layer, 20% dropout and fully-connected dense output layer with softmax output. The embedding and GRU layers have sizes of both 100 for MovieLens and sizes of 400 and 100 for Yoochoose.
- **SASRec:** 1 embedding layer and 1 self-attention layer, 20% dropout and fully-connected dense output layer with softmax output. The embedding sizes and feedforward layer sizes are 64 and 256 for MovieLens and 32 and 512 for Yoochoose.
- **BERT4Rec**: 1 embedding layer and 2 self-attention layers, 20% dropout and fully-connected dense output layer with softmax output. The embedding sizes and feedforward layer sizes are 64 and 128 for MovieLens and 64 and 64 for Yoochoose.

#### 3.2.2 Metrics

To measure the performance of our model we use HitRate@1. HitRate@*k* measures whether the correct item is in the top-*k* position in the recommendation list. HitRate@1 is equivalent to multi-class classification accuracy.

We also make use of two additional metrics to help us analyze the predicted outputs of the models. Given that the LLMs are predicting the item IDs by each single-digit token one by one, we measure the *average deviation* and the *average distance* of the predicted item IDs to the IDs in the input sequence. We measure the average deviation by getting the absolute difference between the predicted ID with each input ID and averaging them. In our experiments where *N* = 5, *y* is the prediction output and *x*_*i*_ is the *i*−*th* item ID in the input sequence, the average deviation is calculated as:


$$\begin{array}{l} \overset{N}{\underset{i}{\sum }}\frac{\left|y-x_{i}\right|}{N} \end{array}$$

The average distance is calculated using the hamming distance between two strings which is the number of positions in the strings in which they differ. For example the strings “1**1**0**5**9” and “1**5**0**6**9” have a hamming distance of 2, pertaining to the two positions that are bolded. The average distance is calculated as:


$$\begin{array}{l} \overset{N}{\underset{i}{\sum }}\frac{Hamming\left(y,x_{i}\right)}{N} \end{array}$$

## 4 Results

We run our experiments five times for each model and dataset combination and the average of the results are presented. We also do one-way ANOVA (Fisher, 1992) followed by Tukey's HSD test (Tukey, 1949) to confirm that the difference in results of the LLMs compared to the baseline methods are statistically significant.

Table 4 shows the HitRate@1 of the LLMs together with the baseline models, along with the average distance and average deviation of their predicted outputs. We see that the LLMs have superior sequential recommendation performance in the MovieLens and Yoochoose datasets under the coldstart setting. With the limited training data, the LLMs were able to take advantage of the closer similarity between IDs in the sequences for its predictions. The baseline algorithms are trying to learn embeddings for each item ID which necessitates more training data to model the user preferences accurately.

**Table 4:** Average distance and average deviation of predictions.

**Dataset**	**Model**	**HitRate@1**	**Average distance**	**Average deviation**
Yoochoose	Danube	**0.0555**	3.50	11,610.96
	Gemma	0.0540	**3.48**	**11,326.80**
	GRU4Rec	0.0440	3.75	13,336.70
	SASRec	0.0354	3.82	13,906.85
	BERT4Rec	0.0499	3.66	12,419.45
MovieLens	Danube	0.0995	3.20	4163.35
	Gemma	**0.1019**	**3.19**	**3,999.54**
	GRU4Rec	0.0934	3.35	4,773.73
	SASRec	0.0898	3.37	5,319.92
	BERT4Rec	0.0854	3.34	4,429.16

Highest HR@1 and the lowest Average Distance and Average Deviation are in bold. The LLMs perform better than the baseline algorithms in the coldstart setting and their predictions are closer to the input sequences both textually and numerically.

Finally, in both datasets the average distance and deviation of the LLMs are smaller than of the baseline models. This suggests that the LLMs are more likely to predict item ID values that are closer in value to the input sequence both textually and numerically. Sequences of IDs that are relatively close to each other are easier for the LLM to learn efficiently.

## 5 Analysis

### 5.1 Tokenization behavior

We analyzed how both Gemma-2B and Danube-1.8B tokenize item IDs. As shown in Table 5 neither model treats numeric item IDs as atomic symbols. Instead, each ID is decomposed into a sequence of digit-level tokens. Because the number of tokens corresponds directly to the number of digits, the models implicitly learn the morphological structure of item IDs rather than their symbolic identity. This explains why the LLMs sometimes predict outputs with similar digit patterns or similar number of digits.

**Table 5:** Example of tokenization output for both Danube and Gemma.

**Model**	**ID**	**Tokenization**
Danube	4,568	[4,5,6,8]
	376	[3,7,7]
Gemma	4,568	[4,5,6,8]
	376	[3,7,7]

However this tokenizer-induced numeric bias cannot fully account for model behavior. Many correct predictions correspond to items that are not numerically close to any input IDs. These predictions cannot be explained by digit continuity or numeric similarity, indicating that the models are also learning co-occurrence structure in the data rather than performing trivial numeric continuations. These observations suggest that small LLMs do exploit digit-level regularities imposed by tokenization but also learn meaningful sequential patterns beyond simple numeric morphology. We show a sample of these predictions from the Danube model in Table 6.

**Table 6:** Sample of correct predictions which are not numerically close to the input IDs.

**Input**	**Prediction**
36,431, 40,655, 40,661, 249, 28,858	30,761
421, 1,183, 205, 143, 117	722
50, 47, 51, 28,413, 9	23,164
2,614, 41,456, 3,735, 6,775, 41,446	2,210

### 5.2 Input Sequence Length

We also ran additional experiments on the Movielens dataset using the Danube-1.8B LLM model and the GRU4Rec basleline model to test the effects of increasing the length of input sequences and show these results in Table 7. Unlike conventional sequential recommenders which typically benefit from longer interaction histories, we find that the small LLM model was not able to take advantage of the longer input sequences to improve its performance significantly, whereas the GRU4Rec model was able to better improve its performance as the input sequence length increased.

**Table 7:** Results of different input sequence lengths on the Movielens dataset.

**Model**	**Input sequence length**	**Tokenization**
Danube	5	0.0995
	10	0.1044
	20	0.0973
	50	0.0917
GRU4Rec	5	0.0934
	10	0.0961
	20	0.1037
	50	**0.1080**

Best result in bold.

Our results show that while the baseline GRU4Rec model can better leverage longer histories through learned item representations, the small LLMs shine at shorter item interaction histories typical of the coldstart scenario in our study.

### 5.3 Inference efficiency

We also measured inference performance for the small LLMs used in our experiments. In our machines,^3^ Gemma-2B achieved a latency of 59.5ms per generated token, while Danube-1.8B achieved 34.3ms, with both models using approximately 5.2GB of GPU memory during inference. Larger LLMs will require much larger computing requirements and also higher latency in the same machines (Chitty-Venkata et al., 2025). Additionally, while these latencies are higher than those of the baseline recommendation models, the LLMs do not require a separate item-embedding matrix whose size grows linearly with the number of items in the catalog. LLMs operate directly on the tokenized string representation of item IDs and therefore rely on a fixed-size tokenizer vocabulary and embedding table. As a result, the memory footprint of the LLMs remains constant regardless of catalog size which can be advantageous in domains where item vocabularies are large or dynamic.

## 6 Conclusion

We have shown that small LLMs can punch above their weight and be viable sequential recommendation models after fine-tuning with LoRA. We also found that the LLMs were more likely to make predictions that are closer to the input sequences both textually and numerically. In our experiments the small LLMs were even able to beat standard sequential recommendation models in the coldstart setting. We also performed token-level analysis, which showed that although the models exploit digit-level tokenization patterns which biases numeric proximity, they also learn meaningful sequential patterns. The LLMs also avoid item-embedding tables and therefore scale independently of catalog size which can be advantageous in domains with extremely large vocabulary sizes. Finally, we also looked at the effects of longer input sequence histories and found that traditional sequential recommenders like GRU4Rec are able to leverage longer item histories better and will eventually beat the small LLMs outside of the coldstart scenario. A future study beyond the scope of this paper could be on how to improve the small LLMs to better leverage longer item histories.

Our results holds great promise in future democratization of the use of small LLMs, which can be run by more organizations with relatively lesser compute capacities. Future work in this field can explore even smaller LLMs, as well as their applicability for other recommendation domains such as ratings predictions. A more systematic study of tokenization strategies such as learned ID embeddings or alternative numeric decomposition methods can be also done for comparison. Using small LLMs for feature augmentation or dataset augmentation can also be additional use cases for exploration. These directions can deepen our understanding of when small LLMs represent a practical alternative to embedding-based recommenders and how they can be used most effectively within real-world systems. We hope that our work also encourages the use of open-source and smaller LLMs in other fields outside the recommendation domain, as they may provide a more realistic alternative than always going for the biggest state-of-the-art models.

## Data Availability

The original contributions presented in the study are included in the article/supplementary material, further inquiries can be directed to the corresponding author.

## Footnotes

1
https://huggingface.co/h2oai/h2o-danube-1.8b-base

2
https://huggingface.co/google/gemma-2b

3 Intel Xeon CPU with 4 NVIDIA GeForce RTX 2080 Ti GPUs.
